# Heart rate and blood pressure response improve the prediction of orthostatic cardiovascular dysregulation in persons with chronic spinal cord injury

**DOI:** 10.14814/phy2.14617

**Published:** 2020-10-20

**Authors:** Siqi Wang, Jill M. Wecht, Bonnie Legg Ditterline, Beatrice Ugiliweneza, Matthew T. Maher, Alexander T. Lombard, Sevda C. Aslan, Alexander V. Ovechkin, Brielle Bethke, Jordan T. H. Gunter, Susan J. Harkema

**Affiliations:** ^1^ Kentucky Spinal Cord Injury Research Center University of Louisville Louisville KY USA; ^2^ Department of Neurological Surgery University of Louisville School of Medicine Louisville KY USA; ^3^ James J Peters VA Medical Center Bronx NY USA; ^4^ Departments of Medicine and Rehabilitation Medicine the Icahn School of Medicine Mount Sinai New York NY USA; ^5^ Kessler Institute for Rehabilitation West Orange NJ USA; ^6^ Kalamazoo College Kalamazoo MI USA; ^7^ Centre College Danville KY USA; ^8^ Frazier Rehab Institute Louisville KY USA

**Keywords:** autonomic nervous system, blood pressure, heart rate, orthostatic hypotension, spinal cord injury

## Abstract

Unstable blood pressure after spinal cord injury (SCI) is not routinely examined but rather predicted by level and completeness of injury (i.e., American Spinal Injury Association Impairment Scale AIS classification). Our aim was to investigate hemodynamic response to a sit‐up test in a large cohort of individuals with chronic SCI to better understand cardiovascular function in this population. Continuous blood pressure and ECG were recorded from individuals with SCI (*n* = 159) and non‐injured individuals (*n* = 48). We found orthostatic hypotension occurred within each level and AIS classification (n = 36). Moreover, 45 individuals with chronic SCI experienced a drop in blood pressure that did not meet the criteria for orthostatic hypotension, but was accompanied by dramatic increases in heart rate, reflecting orthostatic intolerance. A cluster analysis of hemodynamic response to a seated position identified eight distinct patterns of interaction between blood pressure and heart rate during orthostatic stress indicating varied autonomic responses. Algorithmic cluster analysis of heart rate and blood pressure is more sensitive to diagnosing orthostatic cardiovascular dysregulation. This indicates blood pressure instability cannot be predicted by level and completeness of SCI, and the consensus statement definition of orthostatic hypotension is insufficient to characterize the variability of blood pressure and heart rate responses during orthostatic stress. Both blood pressure and heart rate responses are needed to characterize autonomic function after SCI.

## Graphical abstract

The definition of orthostatic hypotension, based solely on an absolute decrease in blood pressure, is insufficient to characterize the adverse blood pressure and heart rate responses during orthostatic stress. Algorithmic cluster analysis of heart rate and blood pressure interaction is more sensitive to diagnosing orthostatic cardiovascular dysregulation. These results demonstrate that cardiovascular instability cannot be predicted by level and completeness of SCI, and both blood pressure and heart rate responses are needed to characterize autonomic dysfunction after SCI.

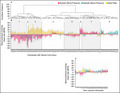

**KEY POINTS**

- Unstable blood pressure after spinal cord injury (SCI) is not routinely examined but rather predicted by the neurological classification of SCI: individuals with high level and complete injury are anticipated to experience more frequent and severe blood pressure instability compared with lower level and incomplete SCI.
- Orthostatic hypotension, that is, a drop of at least 20 mmHg systolic blood pressure or 10 mmHg diastolic blood pressure when assuming an upright position, is a manifestation of this impairment.
- An unexpected finding was that completeness of injury did not predict the incidence of orthostatic hypotension.
- Additionally, the definition of orthostatic hypotension based solely on an absolute decrease in blood pressure is insufficient to characterize the adverse blood pressure and heart rate responses during orthostatic stress.
- Algorithmic cluster analysis of heart rate and blood pressure is more sensitive to diagnosing orthostatic cardiovascular dysregulation.
- These results demonstrate that cardiovascular instability cannot be predicted by level and completeness of SCI, and both blood pressure and heart rate responses are needed to characterize autonomic dysfunction after SCI.

## 1 INTRODUCTION

Severe spinal cord injury (SCI) causes cardiovascular dysregulation resulting in blood pressure instability, which persists throughout life. Persistently low resting blood pressure and heart rate, autonomic dysreflexia, and orthostatic hypotension, among other manifestations (Eldahan & Rabchevsky, 2018; Wecht & Bauman, 2018; Wecht et al., 2013), have been associated with the loss of integrity of spinal sympathetic pathways that facilitate tonic and reflex cardiovascular control (Furlan et al., 2003; Phillips & Krassioukov, 2015). These impairments lead to altered blood pressure responses to upright positioning, delaying therapeutic interventions, and limiting independence and social engagement, thus drastically decreasing health‐related quality of life (Illman et al., 2000; Blackmer, 1997; Carlozzi et al., 2013). Hypotension, even when asymptomatic, adversely impacts the cerebral circulation (Eigenbrodt et al., 2000; Jegede et al., 2010; Nightingale et al., 2020; Wecht & Bauman, 2013; Wu et al., 2012). Identifying those with orthostatic hypotension and unstable blood pressure responses to a postural change is important to improve overall health‐related quality of life and decrease morbidity and mortality of individuals with SCI (Carlozzi et al., 2013; Myers et al., 2007).

Unfortunately, the diagnosis of individuals at risk for conditions like orthostatic hypotension is not a part of routine care (Consortium for Spinal Cord M, 2000; Consortium for Spinal Cord M, 2008). Instead, the risk is most often predicted by the level and severity of injury classified by the International Standards for the Neurological Classification of Spinal Cord Injury, that is, ISNCSCI exam (Kirshblum et al., 2011). Studies have shown that other predictors, such as sympathetic skin responses, should be used to improve the prediction of autonomic function following SCI and that motor and sensory completeness of injury do not correlate with cardiovascular dysregulation (Claydon & Krassioukov, 2006; Sahota et al., 2012). Cervical SCI is commonly associated with more frequent and more severe cardiovascular dysfunction (Claydon & Krassioukov, 2006; Illman et al., 2000; West et al., 2012); however, very few studies have been adequately powered to closely examine cardiovascular responses to orthostasis within similar levels and severities of SCI due to small sample sizes (West et al., 2012, 2013).

Our aim was to quantify the cardiovascular responses (blood pressure and heart rate) to an orthostatic provocation (i.e., the sit‐up test) in a large cohort of individuals with SCI. The sit‐up test was developed for use in populations that are unable to stand (Tang et al., 2012), and reliably induces a physiologically relevant orthostatic stress to induce hypotension (Currie et al., 2015; Shaw et al., 2014). Factors known to affect orthostatic cardiovascular responses in the general population, including age (Franklin & Levy, 2011; Joyner et al., 2015; Stratton et al., 2003), sex (Joyner et al., 2015; Ludwig et al., 2001; Stratton et al., 2003), height (Arvedsen et al., 2012, 2015; Evans et al., 2017), weight (Dewey et al., 2008; Evans et al., 2017), body mass index (Collaboration PS, 2009), and prescribed medications that affect cardiovascular function were considered. Our primary objective was to investigate the prevalence of orthostatic hypotension in the SCI population, as defined by consensus statement developed for use in the non‐injured population ( AAS/AAN, 1996; Freeman et al., 2011) and also used in the SCI population (Krassioukov et al., 2009). Second, we sought to determine the impact of the known factors contributing to orthostatic hypotension in the general population along with injury characteristics (i.e., level and severity of SCI classified by the ISNCSCI exam) on the incidence of orthostatic hypotension in a large cohort of participants with SCI. We hypothesized (a) greater sensory‐motor deficit would not be associated with a greater prevalence of orthostatic hypotension, and (b) factors that affect cardiovascular function in the general population would not relate to the prevalence of orthostatic hypotension nor the blood pressure and heart rate response during the sit‐up test. Our subsequent overall objective was to expand our understanding of the hemodynamic response to a sit‐up test in the SCI population and we hypothesized there would be (a) significant changes in the heart rate that accompany changes in systolic and diastolic blood pressure during orthostatic stress and (b) the restrictive definition of orthostatic hypotension by the consensus statement would be insufficient to identify the prevalence of cardiovascular dysregulation in the SCI population.

## 2 MATERIALS AND METHODS

### 2.1 Participants

This was a cross‐sectional, prospective study conducted at the James J. Peters Veterans Affairs Medical Center (JJP) in Bronx, NY, the Kessler Foundation (KF) in West Orange, NJ, and the Kentucky Spinal Cord Injury Research Center at the University of Louisville (UofL), Louisville, KY. A total of 207 individuals were recruited for assessments including 159 individuals with SCI and 48 age‐matched non‐injured controls. Participants provided a self‐reported medical history and physical examination or received a medical examination onsite. Inclusion criteria for all individuals were: (a) stable medical condition without current illness or infection, (b) greater than 18 years of age, (c) no history of cardiovascular or pulmonary disease unrelated to SCI, Parkinson's disease, or diabetic autonomic neuropathy. Additional inclusion criteria for participants with SCI were: (a) sub‐acute and chronic duration of injury (>6 months), (b) non‐ventilator dependent, and (c) non‐ambulatory. The SCI cohort included 85 individuals with cervical injuries (C2–C8), 21 individuals with high thoracic injuries (T1–T5), and 53 individuals with low cord injuries (T6–L3), with severity classified by the American Spinal Injury Association Impairment Scale (AIS), grade A‐D. Institutional Review Boards at each site reviewed the study protocol and consent documentation for human subject protection and granted annual approval from 2010 to 2018. All participants signed informed consent prior to enrollment in study procedures.

### 2.2 Medications

To limit the variability from the effects of medication on blood pressure and heart rate response to the sit‐up test, medications were stopped for the timespan calculated to approximate five half‐lives, whenever possible (at the discretion of the individuals’ primary care physician), allowing them to be adequately removed from the body. Medications that were requested to be stopped included (a) anticholinergics (including antidepressant, antihistamine, and over‐the‐counter cough and cold medication); (b) sympathomimetics (α‐ and β‐agonists); (c) parasympathomimetic agents; (d) short‐acting α‐ and β‐antagonists; and (e) analgesics including opioids (Zhu et al., 2013). Individuals that reported prescription use of hypertensive or hypotensive medication were tracked to statistically control for chronic effects on hemodynamic response to an upright position.

### 2.3 Study procedures

Each assessment was conducted between 8a.m. and 12p.m. in a quiet, temperature‐controlled room. Participants were instructed to avoid caffeine, alcohol, nicotine, and vigorous exercise for at least 12‐hr prior to testing. Individuals with SCI were asked to empty their bladder upon arrival. Instrumentation was applied supine and included three‐lead ECG (Model RESP 1 with EKG, UFI, Morro Bay, CA, USA; ECG, PowerLab model ML132, ADInstruments, Colorado Springs, CO, USA); respiratory bands or electrodes placed in the 5th intercostal right and left mid‐axillary lines to monitor respiration (Model RESP 1 with EKG, UFI, Morro Bay, CA, USA; Respiratory Belt, UFI or AD Instruments); photoplethysmography to monitor continuous blood pressure from the index finger, middle finger, or thumb (Finapres Medical Systems, Amsterdam, Netherlands); and brachial blood pressure (GE Healthcare, Milwaukee, WI, USA).

Individuals lay in the supine position and, once acclimated, 5 min of resting data were recorded. Individuals were then passively moved into the seated position with the hips and knees at a 90° angle and the back, shoulders, and legs supported with feet placed on a footplate. Beat‐to‐beat blood pressure and heart rate were recorded continuously during the transition and throughout the seated position lasting for 4 min at JJP and KF (SCI: *n* = 83, NI: *n* = 28) and for 15 min at UofL (SCI: *n* = 76, NI: *n* = 20); data collection was on‐going at JJP and KF when UofL was added as a study site and the test duration was lengthened in order to observe the prevalence of delayed orthostatic hypotension. If any research participant had symptoms of pre‐syncope while seated the individual was immediately returned to the supine position and the assessment was terminated. Blood pressure and heart rate signals were sampled at 500 Hz (JJP/KF) or 1,000 Hz (UofL) and stored for offline analysis (JJP/KF: LabVIEW graphical software, National Instruments, Austin, TX, USA; UofL: LabChart, ADInstruments, Colorado Springs, CO, USA). Discrepancies in the sample rate and acquisition software are due to timeline differences between institutions that changed software availability upon data collection.

### 2.4 Data Processing

Finger blood pressure signals were calibrated to brachial blood pressure offline (Silke & McAuley, 1998) using a 2‐point calibration method. Digitized blood pressure and heart rate signals were analyzed with a custom program (MATLAB, the MathWorks, Natick, MA) that performed R‐peak detection of ECG, and peak and trough detection of the blood pressure waveform. Continuous heart rate, systolic blood pressure, and diastolic blood pressure are summarized as the mean of 5‐min in the supine position to estimate resting blood pressure and heart rate, and 1‐min means during the seated position to account for time‐dependent neuronal and hormonal effects to orthostatic stress (Rowell, 1993); change was calculated as the difference between each one‐minute seated mean from the supine mean. Criteria for the incidence of orthostatic hypotension was defined as a decrease in systolic blood pressure of at least 20 mmHg and/or diastolic blood pressure of at least 10 mmHg within 3 min upon the assumption of an upright position, based on the definition established by the American Academy of Neurology and the American Autonomic Society (AAS/AAN, 1996). Orthostatic hypotension was determined from the difference between the lowest mean systolic and/or diastolic blood pressure that occurred within the first 3 min of an upright position and the 5‐min supine mean. Delayed orthostatic hypotension was defined as a fall in blood pressure that met the established criteria that occurred after the first 3 min (Freeman et al., 2011); data on delayed orthostatic hypotension are only available from individuals who were in the upright position for 15 min.

### 2.5 Cluster analysis

We explored cardiovascular response to an upright position to characterize patterns of interaction between systolic blood pressure, diastolic blood pressure, and heart rate changes between the lowest 1‐min mean and the supine mean using agglomerative hierarchical cluster analysis with the Ward's method (Ward, 1963) for the similarity/dissimilarity measure. The ideal number of clusters was obtained using the silhouette method (Rousseeuw, 1987) which incorporates the within‐cluster cohesion and between‐cluster separation. Eight clusters corresponded to a local silhouette coefficient maximum. A dendrogram is used to visualize the clustering process. The cluster analysis was performed in R (R Core Team, 2019) using the function hclust from the stats package.

### 2.6 Statistical analyses

Demographics (age at test, sex, height, weight, and body mass index) and medication use are compared between SCI and non‐injured groups and within SCI subgroups (with and without orthostatic hypotension and across clusters); while age at injury, duration of injury, level of injury and severity of injury (AIS Grade A‐D) are only compared within SCI subgroups (by the incidence of orthostatic hypotension and by cluster). Continuous variables are presented as mean ± standard deviation (*SD*) and compared with the non‐parametric Sign Rank Sum test (2 groups) or Kruskal‐Wallis test (3 or more groups). Categorical variables are summarized as frequency count with percentage and compared using Chi‐square tests or Fisher's exact test. All tests were 2‐tailed with a significance level of 0.05. Statistical analyses were performed in SAS (SAS Institutes, Cary, NC). When the site was added as a covariate to the statistical models, it did not affect the prevalence of orthostatic hypotension nor change the cluster analyses as the identified patterns presented in all centers, indicating the differences between protocols did not affect cardiovascular response to the sit‐up test.

## 3 RESULTS

### 3.1 Research participant characteristics

The adult SCI and non‐injured groups were predominantly male with a wide age range, with the average age at assessment consistent with that reported in most current SCI studies (Table 1). Weight and BMI were similar between groups; height was significantly different between the two groups and was likely associated with the greater proportion of males in the SCI group. In the SCI group, the level of injury spanned all neurological levels, with cervical and low cord representing a greater proportion than high thoracic SCI. All four AIS classifications (AIS A, B, C, and D) were represented; AIS A had the greatest representation, while AIS D had the least representation. Seventeen individuals with SCI reported prescription use of anti‐hypertension or anti‐hypotension medication. None of the NI individuals reported taking anti‐hypertension or anti‐hypotension medication.

**Table 1:** Research participant characteristics

	SCI Individuals			Non‐Injured	
	(*n* = 159)			(*n* = 48)	
Demographics:					
Age at assessment (years)		38 ± 13	40 ± 13		
Age range (years)		19 – 74	21 – 63		
Females, *n* (%)		32 (20%)	16 (33%)		
Males, *n* (%)		127 (80%)	32 (67%)		
Height (cm)		177 ± 10*	172 ±10		
Weight (kg)		82 ± 19	80 ± 18	
BMI (kg/m^2^)		26 ± 6	25 ± 4		
Injury Characteristics:					
Level of injury		C2‐L3	NA		
Cervical *n* (%)		85 (53%)			
High thoracic *n* (%)		21 (13%)			
Low cord *n* (%)		53 (33%)			
Severity of Injury					
AIS A, *n* (%)		60 (38%)	NA		
AIS B, *n* (%)		35 (22%)	NA		
AIS C, *n* (%)		41 (26%)	NA		
AIS D, *n* (%)		23 (14%)	NA		
Age at injury (years)		30 ± 12	NA		
Duration of Injury (years)		9 ± 10	NA		
Medications:					
Anti‐hypertensive, *n* (%)		7 (5%)	0		
Anti‐hypotensive, *n* (%)		10 (7%)	0		

Note

Demographics were compared between SCI and non‐injured individuals. Demographics, age at injury, and duration of injury were summarized with mean ± *SD*; sex, level of injury, severity of injury, and medication use were summarized with frequency count (%).

* *p* < .05 versus Non‐Injured.

### 3.2 Prevalence of orthostatic hypotension: neurological classification of SCI and contributing factors

**Figure 1:**
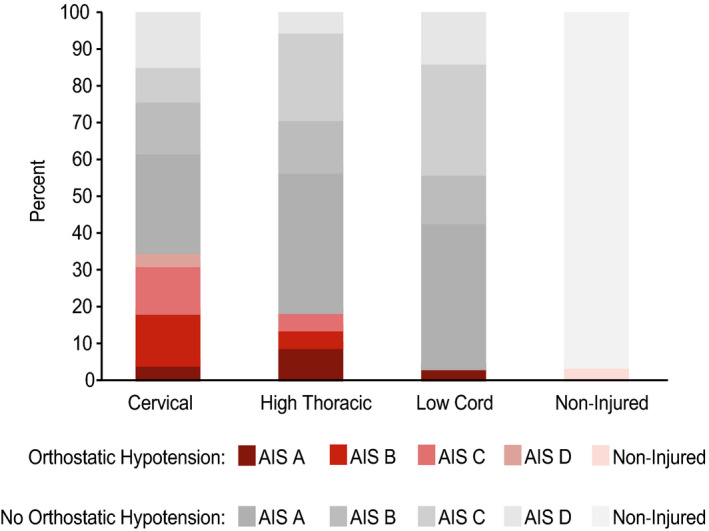
Prevalence of orthostatic hypotension determined by consensus statement (AAS/AAN, 1996; Freeman et al., 2011) by Cervical, High Thoracic, and Low Cord levels and severity of injury (determined by ISNCSCI (Kirshblum et al., 2011). Orthostatic hypotension is defined as a drop in systolic blood pressure (≥20 mmHg) and/or diastolic blood pressure (≥10 mmHg) within 3 minutes of assumption of an upright position. Percentage of individuals that experienced orthostatic hypotension is indicated with shades of red; percentage of individuals that did not experience orthostatic hypotension is indicated with shades of gray. Among cervical SCI, prevalence of orthostatic hypotension was significantly lower in individuals with AIS A classification (5%) compared with AIS B (14%; *p *= 0.007) and AIS C (13%; *p* = 0.002)

The proportion of individuals who met the criteria for orthostatic hypotension as defined by consensus statement (AAS/AAN, 1996; Freeman et al., 2011) in the SCI group was low (36 of 159, or 23%, Figure 1 **,** Table 2), but not as low as compared with non‐injured individuals (2 of 48, 4%). Among cervical SCI, the prevalence of orthostatic hypotension was significantly lower in individuals with AIS A classification (5%) compared with AIS B (14%; *p* = .007) and AIS C (13%; *p* = .002, Figure 1). The overall prevalence of orthostatic hypotension was significantly greater in those with cervical (30 of 85 35%; *p* < .0001) and high thoracic (4 of 21, 19%; *p* = .03) SCI as compared with low cord injury (2 of 53, 4%, Table 2), irrespective of AIS classification. The overall prevalence of orthostatic hypotension was significantly lower in SCI individuals with AIS A classification (8 of 60, 13%) compared with AIS B (13 of 35, 37%; *p* = .007) and AIS C (12 of 41, 29%; *p* = .048, Table 2); the overall prevalence of orthostatic hypotension was not significantly different in AIS A compared with AIS D (3 of 23, 13%). Age at injury and duration of injury was not significantly associated with orthostatic hypotension. Of the 10 individuals with SCI that reported anti‐hypotensive medication use, five (50%) still experienced orthostatic hypotension; none of the seven individuals taking anti‐hypertensive medication experienced orthostatic hypotension. Individuals with SCI that experienced orthostatic hypotension were significantly taller, but was likely associated with the greater proportion of males; otherwise, no demographic characteristics were significantly associated with the prevalence of orthostatic hypotension in individuals with SCI. We monitored for delayed onset of orthostatic hypotension, that is, occurring after the third minute in the upright position (Freeman et al., 2011), in 48% (76 of 159) research participants with SCI and 42% (20 of 48) non‐injured participants; within these subgroups, no non‐injured individuals and only an additional 6 of 76 individuals with SCI experienced delayed onset of orthostatic hypotension.

**Table 2:** Research participant characteristics by the incidence of orthostatic hypotension

	Orthostatic hypotension	
	No (*n* = 123)	Yes (*n* = 36)
Demographics:		
Age at assessment (years)	39 ± 13	37 ± 12
Age range (years)	19 – 74	20 – 61
Females, *n* (%)	28 (23%)	4 (11%)
Males, *n* (%)	95 (77%)	32 (89%)
Height (cm)	176 ± 10	180 ± 9*
Weight (kg)	82 ± 18	81 ± 20
BMI (kg/m^2^)	26 ± 5	25 ± 6
Injury Characteristics:		
Level of injury	C2‐L3	C2‐T10
Cervical, *n* (%)	55 (45%)	30 (83%)†
High thoracic, *n* (%)	17 (14%)	4 (11%)†
Low cord, *n* (%)	51 (41%)	2 (6%)
Severity of Injury		
AIS A, *n* (%)	52 (42%)	8 (22%)
AIS B, *n* (%)	22 (18%)	13 (36%)#
AIS C, *n* (%)	29 (24%)	12 (33%)#
AIS D, *n* (%)	20 (16%)	3 (8%)
Age at injury (years)	30 ± 12	28 ± 13
Duration of injury (years)	9 ± 10	9 ± 9
Medications:		
Anti‐hypertensive, *n* (%)	7 (6%)	0
Anti‐hypotensive, *n* (%)	5 (4%)	5 (14%)

Note

Demographics, injury characteristics, and medication use were compared between SCI individuals with and without orthostatic hypotension. Demographics, age at injury, and duration of injury were summarized with mean ± *SD*; sex, level of injury, severity of injury and medication use were summarized with frequency count (%).

* *p* < .05 versus No orthostatic hypotension;

^†^ *p* < .05 versus low cord;

^#^ *p* < .05 versus AIS A.

### 3.3 Hemodynamic response to the assumption of an upright position: blood pressure and heart rate

**Figure 2:**
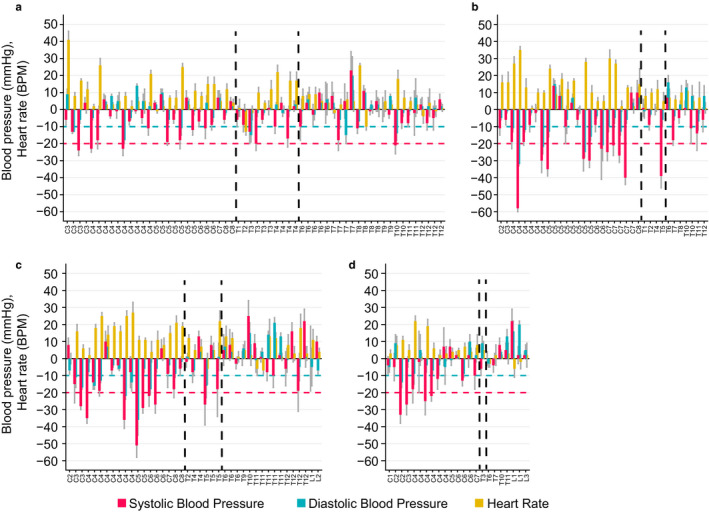
Difference (1‐minute mean ± SD) in systolic blood pressure (mmHg, red vertical bars), diastolic blood pressure (mmHg, green vertical bars), and heart rate (BPM, yellow vertical bars) upon assumption of an upright position in individuals with SCI versus injury level (Panel a: AIS A; Panel b: AIS B; Panel c: AIS C; Panel d: AIS D). Vertical dashed lines delineate Cervical, High Thoracic, and Low Cord Injury groups with injury levels denoted on the x‐axis. Threshold for orthostatic hypotension is illustrated with horizontal dashed lines, defined by consensus statement (AAS/AAN, 1996) as a drop in systolic blood pressure (≥20 mmHg, dashed red line) or diastolic blood pressure (≥10 mmHg, dashed green line) within 3 minutes of assumption of an upright position. Profound blood pressure and heart rate changes occur within all AIS classifications and injury levels with the exception of those with AIS D low cord injury

Dramatic increases in heart rate that accompanied decreases in systolic and diastolic blood pressure after the assumption of an upright position were evident within each level and severity of injury (Figure 2). Individuals with SCI (*n* = 18) that did not meet the criteria for orthostatic hypotension as defined by consensus statement still had a drop in systolic blood pressure between 10 and 20 mmHg and/or a drop in diastolic blood pressure between 5 and 10 mmHg still experienced a robust increase in heart rate (>10 BPM). Those individuals with SCI that did meet the criteria for having orthostatic hypotension also experienced profound increases in heart rate. These adverse blood pressure and heart rate responses occur within all AIS classifications and injury levels except for individuals with AIS D low cord injury.

### 3.4 Hemodynamic response to the assumption of an upright position: cluster analysis

**Figure 3:**
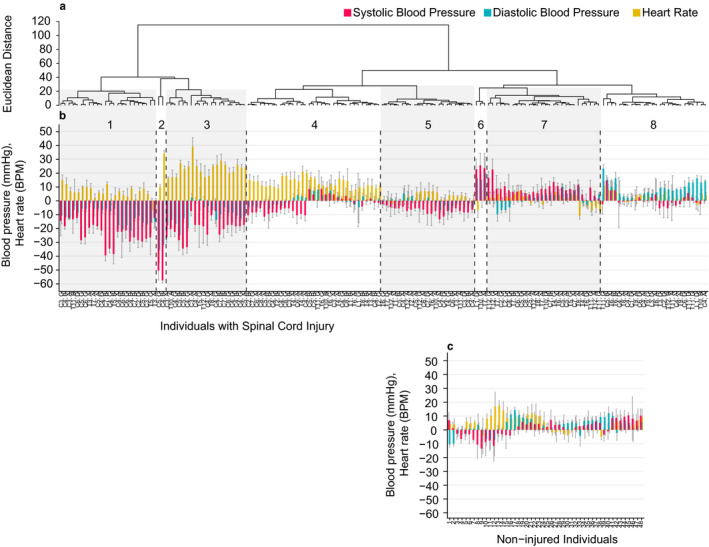
Hierarchical cluster analysis based on change in systolic blood pressure, diastolic blood pressure, and heart rate upon assumption of an upright position. Dendrogram of individuals with SCI (Panel a) is ordered with similar hemodynamic responses having the least Euclidean distance between them. Difference (1‐minute mean ± SD) in systolic blood pressure (mmHg, red vertical bars), diastolic blood pressure (mmHg, green vertical bars), and heart rate (BPM, yellow vertical bars) upon assumption of an upright position were shown for each individual (Panels b and c). Vertical dashed lines delineate between clusters which are denoted as numbers on the top, with injury levels and severities denoted on the x‐axis (Panel b). Each non‐injured individual is denoted on the x‐axis (Panel c)

The hierarchical cluster analysis (Figure 3 **,** Table 3, 4, and 3, 4, 5) demonstrates that there are clusters of systolic, diastolic blood pressure, and heart rate interactions in individuals with SCI. Cluster 1 includes individuals with large decreases in systolic blood pressure (range: −40 to −13 mmHg) and diastolic blood pressures (−22 to −2 mmHg), accompanied by minimal to moderate increases in heart rate (not exceeding 16 BPM). Cluster 2 includes two SCI participants with the largest decreases in systolic (−58, −51 mmHg) and diastolic blood pressures (−36, −32 mmHg); unable to be mitigated with large heart rate changes. Cluster 3 includes individuals with large decreases in SBP (−35 to −8 mmHg) and DBP (−25 to 2 mmHg), but this decrease is accompanied by significantly greater increases in heart rate than Cluster 1 (17 to 40 BPM). Cluster 4 is distinguished by the moderate increases in heart rate (8 to 22 BPM), with lower than Clusters 1–3 or no decreases in systolic and diastolic blood pressures. Cluster 5 includes individuals with lower decreases in systolic (−14 to −3 mmHg) with little to no change in diastolic blood pressure DBP (−7 to 5 mmHg) that was accompanied by little to no change in heart rate (−1 to 8 BPM). Cluster 6 includes individuals with the highest increases in SBP (22 to 25 mmHg) and DBP (15 to 20 mmHg) and minimal changes in HR (−6 to 5 mmHg). Cluster 7 includes individuals with moderate increases in SBP (0 to 22 mmHg) and DBP (−10 to 10 mmHg), accompanied by minimal changes in heart rate. Cluster 8 includes individuals with little to no change in SBP (−6 to 14 mmHg) and moderate increases in DBP (0 to 23 mmHg) accompanied by little to no change in HR (−2 to 12 mmHg). The blood pressure comparisons between clusters were significantly different, with the exception of clusters 1 and 3 (both had great decreases in systolic and diastolic blood pressure) and cluster 4 from 5 to 7 (each had minimal changes in diastolic blood pressure). Heart rate was an important distinguishing factor among the clusters: for example, change in heart rate was significantly different between clusters 1 and 3, while systolic and diastolic pressure was not, potentially indicating different adaptive mechanisms. The reverse relationship is evident between clusters 4 and 5.

**Table 3:** Change in systolic blood pressure upon the assumption of an upright position and comparisons between clusters

	Cluster 1	Cluster 2	Cluster 3	Cluster 4	Cluster 5	Cluster 6	Cluster 7	Cluster 8
	(*n* = 24)	(*n* = 2)	(*n* = 20)	(*n* = 33)	(*n* = 23)	(*n* = 3)	(*n* = 28)	(*n* = 26)
median (range)	−23 (−40, −13)	−55 (−58, −51)	−19 (−35, −8)	−3 (−12, 8)	−7 (−14, −3)	23 (22, 25)	8 (0, 22)	−1 (−6, 14)
Comparison *p*‐values								
Cluster 1	NA	<0.0001	0.0575	<0.0001	<0.0001	<0.0001	<0.0001	<0.0001
Cluster 2		NA	<0.0001	<0.0001	<0.0001	<0.0001	<0.0001	<0.0001
Cluster 3			NA	<0.0001	<0.0001	<0.0001	<0.0001	<0.0001
Cluster 4				NA	0.005	<0.0001	<0.0001	0.0233
Cluster 5					NA	<0.0001	<0.0001	<0.0001
Cluster 6						NA	<0.0001	<0.0001
Cluster 7							NA	<0.0001
Cluster 8								NA

Note

Change in systolic blood pressure (mmHg) is compared between each cluster.

**Table 4:** Change in diastolic blood pressure upon the assumption of an upright position and comparisons between clusters

	Cluster 1	Cluster 2	Cluster 3	Cluster 4	Cluster 5	Cluster 6	Cluster 7	Cluster 8
	(*n* = 24)	(*n* = 2)	(*n* = 20)	(*n* = 33)	(*n* = 23)	(*n* = 3)	(*n* = 28)	(*n* = 26)
median(range)	−11 (−22, −2)	−34 (−36, −32)	−11 (−25, 2)	0 (−6, 8)	−1 (−7, 5)	16 (15, 20)	2 (−10, 10)	8 (0, 23)
Comparison *p*‐values								
Cluster 1	NA	<0.0001	0.4131	<0.0001	<0.0001	<0.0001	<0.0001	<0.0001
Cluster 2		NA	<0.0001	<0.0001	<0.0001	<0.0001	<0.0001	<0.0001
Cluster 3			NA	<0.0001	<0.0001	<0.0001	<0.0001	<0.0001
Cluster 4				NA	0.2653	<0.0001	0.4233	<0.0001
Cluster 5					NA	<0.0001	0.0719	<0.0001
Cluster 6						NA	<0.0001	0.007
Cluster 7							NA	<0.0001
Cluster 8								NA

Note

Change in diastolic blood pressure (mmHg) is compared between each cluster.

**Table 5:** Change in heart rate upon the assumption of an upright position and comparisons between clusters

	Cluster 1	Cluster 2	Cluster 3	Cluster 4	Cluster 5	Cluster 6	Cluster 7	Cluster 8
	(*n* = 24)	(*n* = 2)	(*n* = 20)	(*n* = 33)	(*n* = 23)	(*n* = 3)	(*n* = 28)	(*n* = 26)
median (range)	7 (−6, 16)	23 (11, 35)	25 (17, 40)	13 (8, 22)	4 (−1, 8)	2 (−6, 5)	3 (−10, 8)	4 (−2, 12)
Comparison *p*‐values								
Cluster 1	NA	<0.0001	<0.0001	<0.0001	0.0213	0.0147	<0.0001	0.0293
Cluster 2		NA	0.6843	0.0043	<0.0001	<0.0001	<0.0001	<0.0001
Cluster 3			NA	<0.0001	<0.0001	<0.0001	<0.0001	<0.0001
Cluster 4				NA	<0.0001	<0.0001	<0.0001	<0.0001
Cluster 5					NA	0.1773	0.0419	0.8452
Cluster 6						NA	0.6758	0.1474
Cluster 7							NA	0.0214
Cluster 8								NA

Note

Change in heart rate (BPM) is compared between each cluster.

Although cervical injuries were represented in every cluster except Cluster 6, Clusters 1 and 3 had a significantly greater proportion of individuals with cervical injuries and a significantly smaller proportion of individuals with lower cord injuries when compared with Clusters 7 and 8 (Table 6). Classification AIS A was also represented in all cluster groups except for Cluster 2. The proportion of individuals in Cluster 5 with AIS A classification was significantly greater compared with Cluster 1. Classification AIS C was present in every cluster and AIS D in every cluster with the exception of Clusters 2 and 4. Orthostatic hypotension as defined by the consensus statement was present in clusters 1 (20 of 24, 83%), 2 (2 of 2, 100%), 3 (13 of 20, 65%), and 7 (1 of 28, 4%).

**Table 6:** Research participant characteristics by cluster

		Cluster 1	Cluster 2	Cluster 3	Cluster 4	Cluster 5	Cluster 6	Cluster 7	Cluster 8	Significantly different clusters (*p*‐value < 0.05)											
		(*n* = 24)	(*n* = 2)	(*n* = 20)	(*n* = 33)	(*n* = 23)	(*n* = 3)	(*n* = 28)	(*n* = 26)												
Demographics:																					
Age at test (years)		39 ± 11	31 ± 13	34 ± 11	31 ± 10	44 ± 14	38 ± 12	28 ± 43	42 ± 14	4&1,	5&3,	7&3,	8&3,	5&4,	7&4,	8&4					
Age Range (years)		23 – 61	21 – 40	19 – 57	19 – 55	21 – 72	28 – 51	19 – 74	23 – 71												
Females *n* (%)		3 (13%)	0 (0%)	4 (20%)	10 (30%)	6 (26%)	0 (0%)	5 (18%)	4 (15%)												
Height (cm)		180 ± 8	183 ± 4	180 ± 10	176 ± 11	174 ± 10	174 ± 12	28 ± 175	178 ± 10	5&1,	5&3,	7&3,									
Weight (kg)		84 ± 24	88 ± 5	73 ± 12	75 ± 18	84 ± 22	89 ± 21	28 ± 81	90 ± 15	3&1,	8&3,	8&4,									
BMI (kg/m^2^)		26 ± 7	26 ± 0	22 ± 3	24 ± 5	27 ± 6	29 ± 5	28 ± 27	28 ± 4	3&1,	5&3,	6&3,	7&3,	8&3,	5&4,	8&4,					
Orthostatic Hypotension:																					
no, (%)		4 (17%)	0 (0%)	7 (35%)	33 (100%)	23 (100%)	3 (100%)	27 (96%)	26 (100%)	3&1,	7&1,	4&2,	5&2,	6&2,	8&2,	5&4,	6&4,	8&4,	6&5,	8&5,	8&6
yes, (%)		20 (83%)	2 (100%)	13 (65%)	0 (0%)	0 (0%)	0 (0%)	1 (4%)	0 (0%)												
Injury Characteristics:																					
Level of injury																					
Cervical *n* (%)		18 (75%)	2 (100%)	16 (80%)	18 (55%)	11 (48%)	0 (0%)	10 (36%)	10 (38%)	7&1,	8&1,	7&3,	8&3								
High thoracic *n* (%)		4 (17%)	0 (0%)	1 (5%)	8 (24%)	4 (17%)	0 (0%)	3 (11%)	1 (4%)												
Low cord *n* (%)		2 (8%)	0 (0%)	3 (15%)	7 (21%)	8 (35%)	3 (100%)	15 (54%)	15 (58%)	7&1,	8&1,	7&3,	8&3,	7&4,	8&4						
Age at injury (years)		28 ± 12	27 ± 15	27 ± 12	25 ± 8	35 ± 14	27 ± 6	28 ± 29	35 ± 14	5&1,	8&1,	5&3,	8&3,	5&4,	8&4						
Duration of Injury (years)		11 ± 10	3 ± 2	7 ± 6	6 ± 8	9 ± 10	11 ± 9	28 ± 14	6 ± 7	4&1,	7&3,	7&4,	8&7								
	A, *n* (%)	6 (25%)	0 (0%)	6 (30%)	11 (33%)	13 (57%)	1 (33%)	13 (46%)	10 (38%)	5&1											
AIS	B, *n* (%)	8 (33%)	1 (50%)	5 (25%)	12 (36%)	3 (13%)	0 (0%)	2 (7%)	4 (15%)												
	C, *n* (%)	7 (29%)	1 (50%)	7 (35%)	10 (30%)	3 (13%)	1 (33%)	9 (32%)	3 (12%)												
	D, *n* (%)	3 (13%)	0 (0%)	2 (10%)	0 (0%)	4 (17%)	1 (33%)	4 (14%)	9 (35%)	2&1,	3&2,	5&2,	6&2,	7&2,	8&2						
Medications:																					
Anti‐hypertensive, *n* (%)		0 (0%)	0 (0%)	1 (5%)	0 (0%)	1 (5%)	0 (0%)	2 (7%)	3 (13%)	3&2,	5&2,	7&2,	8&2								
Anti‐hypotensive, *n* (%)		3 (13%)	0 (0%)	2 (10%)	1 (3%)	1 (5%)	0 (0%)	3 (11%)	0 (0%)	2&1,	3&2,	4&2,	5&2,	7&2							

Note

Demographics, the occurrence of orthostatic hypotension defined by the consensus statement, injury characteristics, and medication use were compared between clusters. Demographics, age at injury, and duration of injury were summarized with mean ± *SD*; sex, occurrence of orthostatic hypotension, level of injury, and severity of injury were summarized with frequency count (%).

## 4 DISCUSSION

The prevalence of orthostatic hypotension as defined by the consensus statement (Freeman et al., 2011) in chronic SCI individuals (*n* = 159) who are non‐ambulatory is low (23%) and not related to the severity of their sensory‐motor deficits, although higher in those with cervical and high thoracic injuries compared with low cord injuries. To date, this is the largest cohort of individuals with chronic SCI who are non‐ambulatory to assess cardiovascular orthostatic regulation in response to a sit‐up test. Additionally, factors known to affect cardiovascular dysregulation in the general population were not significantly associated with orthostatic hypotension in individuals with SCI. Dramatic increases in heart rate accompanied decreases in systolic and diastolic blood pressure upon the assumption of an upright position within each level and severity of the injury group, indicating a much more profound cardiovascular deficit than discovered solely by the restrictive definition of orthostatic hypotension as defined by the consensus statement. When considering the interrelationship between systolic blood pressure, diastolic blood pressure, and heart rate upon the assumption of an upright position with hierarchical cluster analysis, we found delineated hemodynamic responses that more effectively identify the prevalence of cardiovascular dysregulation in the chronic SCI population. Further, characterization of cardiovascular orthostatic dysregulation based solely on the threshold of systolic blood pressure and/or diastolic blood pressure falls, as in the consensus statement on the definition of orthostatic hypotension, does not fully represent the degree of hemodynamic instability that occurs in chronic SCI. The diverse patterns of blood pressure and heart rate response to orthostasis in individuals with chronic injury suggests that the interaction between blood pressure and heart rate should be considered in understanding autonomic dysfunction after SCI and its consequences on morbidity and quality of life.

### 4.1 Prevalence of orthostatic hypotension by level and severity of injury

Neurological level of injury, as assessed on the ISNCSCI exam, is associated with a higher degree of cardiovascular dysfunction after chronic SCI, and several reports indicate a higher prevalence of orthostatic hypotension (as defined by consensus statement) in individuals with cervical SCI compared with thoracic SCI (Claydon & Krassioukov, 2006; Sahota et al., 2012; Sisto et al., 2012). However, we found decreased systolic blood pressure upon the assumption of an upright position, even those that met the criteria for orthostatic hypotension, was not limited to individuals with cervical injuries and occurred within each injury and AIS classification. Moreover, individuals with cervical SCI classified as AIS B and C were significantly more likely to have orthostatic hypotension than cervical AIS A, and two participants with low cord injury met the criteria for the diagnosis of orthostatic hypotension. Deconditioning due to immobility and decreases in venous return secondary to inactivity of the skeletal muscle pump can occur in all levels of SCI, irrespective of demographic characteristics. Furthermore, innervation of splanchnic and lower extremity vasculature arises from sympathetic neurons between spinal segments T6–L2. These factors and other, yet unstudied factors, may be important contributors to blood pressure control during orthostasis and could explain why individuals with thoracic and low cord injuries still experience orthostatic hypotension upon the assumption of an upright position.

In contrast to the neurological level of injury, our findings indicate a minimal association between the sparing of motor‐sensory pathways and cardiovascular response to the sit‐up test. We found a significantly lower prevalence of orthostatic hypotension in those with cervical AIS A injuries compared with cervical AIS B and C injuries, and no difference between the overall prevalence of orthostatic hypotension in AIS A compared with AIS D. Anatomical locations of descending dorsolateral vasomotor pathways can vary (Furlan et al., 2003) and may not be in proximity to spinothalamic and corticospinal tracts of the sensory and motor pathways used to determine AIS classification. In addition, local sympathetic veno‐arteriolar reflexes may contribute to the maintenance of orthostatic blood pressure, and these reflexes are mediated independently of supraspinal control (Henriksen, 1977; Henriksen & Sejrsen, 1977). AIS classification does not accurately predict the incidence of orthostatic hypotension after SCI, and previous studies demonstrate other predictors (e.g., sympathetic skin response) correlate with cardiovascular dysregulation better than motor‐sensory completeness of injury (Claydon & Krassioukov, 2006; Lucci et al., 2020; Sahota et al., 2012).

Autonomic dysregulation is unrecognized and widely untreated in the chronic SCI population—many individuals living with SCI report living with persistent feelings of fatigue but do not attribute this to chronic hypotension (Bozzini et al., 2018), while events like orthostatic hypotension and autonomic dysreflexia can occur without any symptoms until they are severe enough to require medical intervention (Phillips & Krassioukov, 2015). As a result, there is a significant disparity between the prevalence of chronic blood pressure instability in SCI and its treatment (Zhu et al., 2013). There is also mounting evidence that, even when asymptomatic, chronic blood pressure instability can impair the ability of the cerebral vasculature to buffer blood pressure (Saleem et al., 2018) and dramatically increase the risk of stroke (Eigenbrodt et al., 2000; Wu et al., 2012), increase arterial stiffness (Currie et al., 2019) and cause cognitive impairment (Jegede et al., 2010; Sachdeva et al., 2019; Wecht & Bauman, 2013; Wecht et al., 2012), leading to significant cardiovascular morbidity and mortality. Therefore, a valid method to assess and quantify the degree of cardiovascular autonomic impairment is needed for the goal of reducing cardiovascular morbidity and mortality and improving health‐related quality of life after SCI.

### 4.2 Pattern of interactions between blood pressure and heart rate

The distinct clusters that described the varied hemodynamic responses to the challenge of an upright position with more details than the consensus definition of orthostatic hypotension indicate different maladaptations to cardiovascular control after chronic SCI. Clusters 1, 2, and 3 had the largest decreases in systolic and diastolic blood pressures but were distinguished by Cluster 1 having significantly lower increases in heart rate. This possibly indicates a differential functional ability of the parasympathetic system to respond to those individuals. In Clusters 2 and 3, parasympathetic withdrawal was robust; however, unable to compensate for impaired sympathetic vasomotor control. Cluster 4 shows vagal withdrawal leading to significant increases in heart rate was able to substantially mitigate, and in some cases complete arrest, orthostatic hypotension. Cluster 5 appears to reflect a more normal autonomic control of heart rate and blood pressure responses and includes all AIS levels and classifications in this group. Clusters 6 and 7 had increases in systolic blood pressure without significant changes in heart rate, potentially adaptations to compensate for the low blood pressure that may have resulted from spasticity or, possibly, autonomic dysreflexia even though triggers (i.e., cutaneous, bowel, or bladder stimuli) were identified and mitigated before and throughout each assessment. Given the orthostatic provocation and the fact that the increases in blood pressure were largely in participants with lesions below T6, these responses may reflect an emerging phenomenon, orthostatic hypertension, which may have adverse effects on cardiovascular health (Fessel & Robertson, 2006; Magkas et al., 2019; Wecht et al., 2016). Cluster 8 was differentiated by increases in diastolic pressures whose mechanisms are unclear.

The understanding of cardiovascular dysfunction by the diagnosis of orthostatic hypotension defined by the consensus statement is not sufficient to fully characterize the prevalence or the presentation of hemodynamic responses. Moreover, including heart rate in these interactions lends insight into autonomic control of the entire cardiovascular system after SCI, not just blood pressure regulation. Patterns of blood pressure and heart rate response have been reported in non‐injured individuals with idiopathic orthostatic hypotension (Streeten et al., 1988), and five distinct patterns of systolic blood pressure, diastolic blood pressure, and heart rate response to standing (i.e., orthostatic stress) were identified. Further, prediction of cardiovascular maladaptation with chronic spinal cord injury cannot be associated with the level or severity of injury based on motor and sensory clinical assessments.

### 4.3 Cardiovascular orthostatic responses

Factors that are predictive of orthostatic hypotension in the general population, including age at test, sex, body mass index, and the number of prescribed medications, did not significantly predict the cardiovascular hemodynamic responses to orthostatic stress in our individuals with SCI. While the level of injury did discriminate between cluster groups describing heart rate and blood pressure responses to the sit‐up test, AIS classification, and time since injury did not. A previous report examined blood pressure change in response to the sit‐up test in 52 Paralympic athletes found a significant univariate relationship with the level of neurological injury (West et al., 2014); however, the addition of sympathetic skin response (i.e., autonomic integrity) significantly improved prediction models. We recognize that the assessment of sympathetic skin response may be used to quantify autonomic function after SCI to ascertain the degree of cardiovascular impairment, but it is not part of routine clinical practice. The most recent version of the International Standards to document remaining Autonomic Function after SCI (ISAFSCI), published in 2012 (Krassioukov et al., 2012), includes only clinical records of supine hypotension or hypertension at the time of assessment, but we found that supine blood pressure had a minimal effect on hemodynamic response to an upright position. Clearly a valid, sensitive, and clinically useful tool that can be used to assess the degree of cardiovascular autonomic impairment after SCI is needed.

### 4.4 Clinical significance

There are multiple redundant mechanisms of blood pressure regulation during orthostatic stress (Rowell, 1993). The patterns of blood pressure and heart rate responses during orthostatic stress in individuals with chronic SCI likely result from one or more of these mechanisms, which include neural, hormonal, cardiac, and vascular alterations. Understanding how the cardiovascular system responds to a clinically relevant orthostatic stress—the sit‐up test—can significantly improve diagnostic practices to guide clinical care and mitigate the long‐term adverse consequences of autonomic nervous system impairment in the SCI population. At present, there is a paucity of safe and effective treatment options for cardiovascular autonomic impairment for clinical use in the SCI population. Increasing the clinical armamentarium of safe and effective treatment options for hypotension, orthostatic hypotension, and autonomic dysreflexia should be a priority. In order to do this, one must recognize the heterogeneity of cardiovascular orthostatic responses in the SCI population beyond the limited definition of orthostatic hypotension, and that these responses are independent of the level and severity of injury. This algorithmic approach to classify cardiovascular autonomic impairment with orthostatic challenge could be utilized to better diagnose those with chronic SCI.

### 4.5 Limitations

There are a few study limitations that should be considered when interpreting the results presented. First, 1‐min averages of seated blood pressure and heart rate are reported, but we did not consider beat‐to‐beat blood pressure or heart rate variability which might lend insight into the contribution of autonomic neural control to these cardiovascular orthostatic responses. Second, the prevalence of delayed orthostatic hypotension was evaluated in a subset of participants who were tested beyond 3‐min and was present in 25% of those who experienced orthostatic hypotension; therefore, more attention should be paid to the prevalence and clinical significance of delayed orthostatic hypotension in the SCI population. Third, although this was a relatively large, heterogeneous group of participants with SCI, future study should be designed to determine the influence of modifiable factors such as exercise, diet, medical conditions, and use of drugs and alcohol on cardiovascular hemodynamic responses to orthostasis. Fourth, we did not evaluate the degree of the autonomic function using the ISAFSCI, which would have provided a subjective correlate of baseline cardiovascular hemodynamic compromise. Finally, although the sit‐up test is a clinically relevant orthostatic stressor, the sensitivity and repeatability of this assessment to evaluate cardiovascular autonomic integrity following SCI warrants further study.

### 4.6 Conclusions

Evaluation of sensorimotor function using the ISNCSCI exam does not provide enough information to describe the remaining autonomic nervous system function following SCI. Furthermore, the occurrence of orthostatic hypotension, by the definition in the consensus statement, cannot be used exclusively to ascertain the degree of cardiovascular autonomic impairment in individuals with SCI. Cluster analysis of blood pressure and heart rate responses to orthostatic stress identified eight distinct patterns that could not be fully explained by the level and severity of injury or contributing factors known to affect orthostatic cardiovascular responses in non‐injured population. Cardiovascular orthostatic dysregulation in persons with chronic SCI is highly heterogeneous and variations among individual patients should be assessed in the clinical setting independent of—but concomitant with—neurological classification of SCI.

## CONFLICT OF INTEREST

The authors have no conflict of interest to declare.

## AUTHOR CONTRIBUTIONS

SJH and JMW designed the study and provided oversight for data acquisition and data analyses. SW, JMW, BLD, SCA, MTM, ATL, AVO, BB, JTHG, and SJH performed data acquisition and data analysis. BU created the statistical design and performed statistical analyses. SW, SJH, BLD, and JMW collaborated on the interpretation of the data. All authors were involved in manuscript preparation and approved the final manuscript for submission.

## Data Availability

Data reported in this manuscript will be made available upon reasonable request.
